# Safety and efficacy of glecaprevir/pibrentasvir for the treatment of chronic hepatitis C in patients aged 65 years or older

**DOI:** 10.1371/journal.pone.0208506

**Published:** 2019-01-02

**Authors:** Graham R. Foster, Tarik Asselah, Sarah Kopecky-Bromberg, Yang Lei, Armen Asatryan, Roger Trinh, Neddie Zadeikis, Federico J. Mensa

**Affiliations:** 1 Barts Liver Centre, Blizard Institute, Queen Mary University of London, London, United Kingdom; 2 Hepatology Department, UMR1149, Physiopathology and Treatment of Viral Hepatitis, Centre de Recherche sur l’Inflammation and Université Denis Diderot Paris 7, Beaujon Hospital, AP-HP, Clichy, France; 3 AbbVie Inc., North Chicago, Illinois, United States of America; Kaohsiung Medical University, TAIWAN

## Abstract

Finding safe and effective treatments for chronic hepatitis C virus (HCV) infection in the elderly is of clinical interest given the comorbidities and associated polypharmacy in this population. However, the number of patients older than age 65 years enrolled into clinical trials of anti-HCV medications generally have been limited and thus reaching meaningful conclusions for this demographic has been difficult. Glecaprevir/pibrentasvir is a once-daily, all-oral, ribavirin-free, pangenotypic direct-acting antiviral (DAA) combination therapy that has demonstrated high sustained virologic response rates at post-treatment week 12 (SVR12) and a favorable safety profile in patients with chronic HCV infection. This analysis evaluated the safety and efficacy of glecaprevir/pibrentasvir in patients aged ≥65 years. Data were pooled for treatment-naïve and -experienced patients with chronic HCV genotype (GT) 1–6 infections who received glecaprevir/pibrentasvir for 8, 12, or 16 weeks in 9 Phase 2 and 3 trials. SVR12 and adverse events (AEs) were evaluated for patients aged ≥65 versus <65 years. Of the 2369 patients enrolled, 328 (14%) were aged ≥65 years. Among patients aged ≥65 years, 42% and 34% had GT1 and GT2, respectively; 40% were treatment-experienced and 20% had compensated cirrhosis. Glecaprevir/pibrentasvir treatment resulted in SVR12 rates of 97.9% (95% CI, 96.3–99.4; n/N = 321/328) for patients aged ≥65 years and 97.3% (95% CI, 96.6–98.0; n/N = 1986/2041) for patients aged <65 years. The rates were not significantly different between the two age groups (*P* = 0.555). DAA-related AEs leading to treatment discontinuation, or serious AEs were similarly rare (<0.5%) for patients ≥65 and <65 years old. Glecaprevir/pibrentasvir is an efficacious and well-tolerated treatment option for patients aged ≥65 years with chronic HCV infection.

## Introduction

The age of the global population of patients with chronic hepatitis C virus (HCV) infection is gradually increasing, in particular in the United States, Japan, and Italy [1–5]. As patients enter older age, it is important to understand the impact of age on the safety and efficacy of current HCV antiviral therapies. However, patients of advanced age have typically been under-represented in clinical trials of anti-HCV therapies and robust data are lacking for this patient group [6, 7].

Elderly patients historically have been considered difficult to treat [6]. Older age is associated with a greater prevalence of advanced fibrosis, more rapid fibrosis progression and cirrhosis, and a greater risk of liver cancer than younger age [6, 7]. Comorbidities that require the frequent use of concomitant medications are more common in elderly patients, compared with younger patients, and therefore there is a potential risk of exposure to drug–drug interactions. [8]. New, all-oral direct-acting antivirals (DAAs) have a much better tolerability profile compared with previous interferon (IFN)-based regimens [7]. Furthermore, findings in real-world studies support that DAAs have high efficacy, safety, and tolerability in elderly patients [9–12]. However, elderly patients are frequently excluded from clinical trials of DAA efficacy and safety [13].

Glecaprevir/pibrentasvir is a once-daily, all-oral, ribavirin (RBV)-free, pangenotypic, DAA combination therapy that has shown high rates of sustained virologic response at post-treatment week 12 (SVR12) and a favorable safety profile in patients with chronic HCV infection, including patients with compensated cirrhosis, prior treatment failures to IFN-based and DAA-containing regimens, severe kidney impairment including dialysis, patients who are post liver or kidney transplant [14], or those with human immunodeficiency virus (HIV) coinfection [15–23]. To evaluate the safety and efficacy of glecaprevir/pibrentasvir in elderly patients, an analysis of integrated data from patients aged 65 years or older enrolled in glecaprevir/pibrentasvir’s registrational clinical trial program was conducted. There was no upper age limit for enrollment into the program.

## Methods

This is an analysis using data integrated from patients who received glecaprevir 300 mg and pibrentasvir 120 mg enrolled in registrational clinical trials of glecaprevir/pibrentasvir for the treatment of chronic HCV infections. These comprise 9 Phase 2 and 3, multicenter, clinical studies: MAGELLAN-1 (NCT02446717) [20, 22], SURVEYOR-I and -II (NCT02243280 and NCT02243293) [15, 18, 19, 21], ENDURANCE-1 (NCT02604017) [23], ENDURANCE-2 (NCT02640482) [15], ENDURANCE-3 (NCT02640157) [23], ENDURANCE-4 (NCT02636595) [15], EXPEDITION-1 (NCT02642432) [16], and EXPEDITION-4 (NCT02651194) [17].

The protocols, designed and sponsored by AbbVie Inc., Chicago, IL, for the original trials were approved by the independent ethics committee or institutional review board for each trial center. The trials were conducted in accordance with the Good Clinical Practice Guidelines and the ethical principles of the Declaration of Helsinki, and all patients provided written informed consent. All authors had access to trial data and participated in the writing, review, and approval of the final manuscript. All trial data were fully anonymized prior to access by the authors. For trials included in this analysis, Dr Foster and Dr Asselah were Principal Investigators; Dr Armen Asatryan and Dr Neddie Zadeikis were Lead Medical Directors; Dr Yang Lei was the Clinical Statistician; and Dr Sarah Kopecky-Bromberg was the Clinical Scientist. Dr Roger Trinh and Dr Federico Mensa designed the protocols and provided study oversight for these trials as well as the glecaprevir/pibrentasvir clinical program.

### Patients

The trials enrolled patients aged ≥18 years with no upper age limit with chronic HCV genotype (GT) 1–6 infections (HCV RNA ≥1000 IU/mL). HCV GT at baseline was determined by the central laboratory; for efficacy analyses, HCV GT was determined by phylogenetic analysis (or Sanger sequencing if phylogenetic analysis was not available). Patients were either non-cirrhotic or had compensated cirrhosis. Absence of cirrhosis was confirmed by liver biopsy, transient elastography (FibroScan [Echosens, Paris, France] <12.5 kPa), or serum markers (FibroTest [BioPredictive, Paris, France] ≤0.48 and aminotransferase-to-platelet ratio index [APRI] <1). Presence of cirrhosis was documented by a liver biopsy with a METAVIR (or equivalent) fibrosis score of 3 or Ishak fibrosis score >4; FibroTest score ≥0.75 with APRI >2, or FibroScan ≥14.6 kPa; and patients must have had a Child–Pugh score of ≤6 at screening and no current or past evidence of Child–Pugh B or C classification or clinical history of liver decompensation. Patients with indeterminate FibroScan were required to have liver biopsy, and patients with indeterminate FibroTest or conflicting FibroTest and APRI scores were required to have transient elastography or liver biopsy to determine cirrhosis status. The elderly population for this analysis was defined as patients aged 65 years or older.

Patients were HCV treatment-naïve or had failed previous HCV therapy with IFN ± RBV, pegylated IFN ± RBV, or sofosbuvir + RBV ± pegylated IFN. Patients in the MAGELLAN-1 trial had completed treatment with a DAA-containing regimen (NS5A inhibitor with or without protease inhibitor) with an outcome of on-treatment failure or post-treatment relapse [20, 22]. Patients coinfected with hepatitis B virus, or with more than one HCV GT were excluded. ENDURANCE-1 included patients with HIV coinfection [23] and EXPEDITION-4 was a dedicated study conducted in patients with severe renal impairment, including those undergoing dialysis [17].

### Treatments

All patients received glecaprevir 300 mg and pibrentasvir 120 mg once daily (dosed as separate medications or as 3 coformulated 100 mg/40 mg oral tablets taken once daily with food) for 8, 12, or 16 weeks.

### Outcomes and statistical analyses

The primary endpoint for each trial was the percentage of patients who achieved SVR12 (HCV RNA <15 IU/mL) in the intention-to-treat (ITT) population, defined as all randomized patients who received at least 1 dose of study drug. Both the COBAS TaqMan HCV Test (v2.0) and COBAS AmpliPrep/COBAS TaqMan HCV Test (v2.0) kits (Roche Molecular Diagnostics) were used across the studies in this analysis to determine HCV RNA concentrations. Efficacy was analyzed using data pooled for patients aged ≥65 years or <65 years. Two-sided 95% confidence intervals (CIs) for SVR12 rates were calculated using the normal approximation to the binomial distribution. Backward imputation was used to impute missing SVR12 data, where applicable. Otherwise, patients with missing data were counted as treatment failures. The difference in the overall SVR12 rates between the two age groups was analyzed using a two-tailed Fisher’s exact test. SVR12 was also analyzed for the modified ITT (mITT) population, defined as the ITT population excluding patients who did not achieve SVR12 for reasons other than virologic failure. Patients who had low compliance but stayed in the study were not excluded in this mITT. Adverse events (AEs; Medical Dictionary for Regulatory Activities version 19.0) and laboratory abnormalities were monitored and recorded throughout the studies and are summarized using descriptive statistics. Because most of the clinical trials excluded patients with an estimated creatinine clearance of <50 mL/minute, safety outcomes were evaluated separately for patients with and without severe renal impairment as it would be expected that patients with severe renal impairment would have a different safety profile from those without severe renal impairment.

## Results

### Patients

A total of 2369 patients were included in this analysis: 328 patients aged ≥65 years, 2041 patients aged <65 years, and 47 patients aged ≥75 years (Table 1). Most elderly patients were white (68%), HCV treatment-naïve (60%), and did not have cirrhosis (80%). The majority of elderly patients received either 8 weeks (29%) or 12 weeks (65%) of treatment with glecaprevir/pibrentasvir. Compared with non-elderly patients, patients in the elderly cohort tended to have a greater frequency of HCV GT2 (i.e., 34% vs 18%) and HCV GT5 infections (i.e. 4% vs 1%), and less frequency of HCV GT3 infections (i.e., 11% vs 30%; Table 1). As expected, prevalence of some comorbidities tended to be greater for elderly patients than for younger patients, such as cirrhosis (20% vs 12%), diabetes (17% vs 7%), hypertension (54% vs 23%), cardiovascular disease (62% vs 28%), and severe renal impairment (estimated glomerular filtration rate <30 mL/min/1.73 m^2^; 9% vs 4%). Non-elderly patients reported a greater frequency of bipolar disorder or depression compared with elderly patients (22% vs 16%). Elderly patients were also more likely to be receiving some concomitant medications corresponding to the increased prevalence of reported comorbidities in this population, including antihypertensive (45% vs 18%), diuretic (15% vs 6%), and lipid-lowering medications (15% vs 7%; Table 2; see S1 Table for concomitant medications in patients with severe renal impairment). The percentage of patients receiving antacids and proton pump inhibitors, and antidepressants, were similar between elderly and non-elderly patients (19% vs 15% and 14% vs 16%, respectively). Overall rates of compliance were similar between the two age groups (88% vs 90%; Table 1). For patients who were receiving 4 or more concomitant medications in addition to glecaprevir/pibrentasvir, 84% of elderly patients (n/N = 163/195) were compliant with the study drug versus 89% of non-elderly patients (n/N = 708/797; see S2 Table).

**Table 1:** Baseline demographics and clinical characteristics.

Characteristics	Patients Aged ≥65 Years (n = 328)	Patients Aged <65 Years (n = 2041)	Total (N = 2369)	*P*-value^f^
Female, n (%)	149 (45)	902 (44)	1051 (44)	0.677
Race, n (%)				
White	223 (68)	1675 (82)	1898 (80)	<0.001
Black	33 (10)	116 (6)	149 (6)	0.002
Asian	68 (21)	204 (10)	272 (11)	<0.001
Other^a^	4 (1)	46 (2)	50 (2)	
Hispanic or Latino ethnicity, n (%)	31 (9)	180 (9)	211 (9)	0.709
Age, mean ± SD (range), years	69.3 ± 4.3 (65–88)	49.8 ± 10.4 (19–64)	52.5 ± 11.8 (19–88)	<0.001
≥75 years, n (%)	47 (14)	–	47 (2)	<0.001
Body mass index, mean (SD), kg/m^2^	26.5 (4.9)	26.7 (5.1)	26.7 (5.1)	0.476
≥30 kg/m^2^, n (%)	66 (20)	436 (21)	502 (21)	0.610
HCV genotype, n (%)				
GT1	139 (42)	848 (42)	987 (42)	0.777
GT2	111 (34)	366 (18)	477 (20)	<0.001
GT3	37 (11)	606 (30)	643 (27)	<0.001
GT4	24 (7)	158 (8)	182 (8)	0.789
GT5	12 (4)	20 (1)	32 (1)	<0.001
GT6	5 (2)	43 (2)	48 (2)	0.487
*IL28B* genotype, n (%)^b^				
CC	113 (35)	653 (32)	766 (32)	0.361
CT	155 (47)	1069 (52)	1224 (52)	0.093
TT	59 (18)	318 (16)	377 (16)	0.260
HCV RNA, mean (SD), log_10_ IU/mL	6.1 (0.9)	6.1 (0.8)	6.1 (0.8)	0.066
HCV RNA, n (%)				
≥1 million IU/mL	200 (61)	1207 (59)	1407 (59)	0.529
≥6 million IU/mL	60 (18)	457 (22)	517 (22)	0.095
Treatment history, n (%)				
Treatment-naïve	198 (60)	1442 (71)	1640 (69)	<0.001
Treatment-experienced	130 (40)	599 (29)	729 (31)	<0.001
IFN/pegIFN ± RBV ± sofosbuvir	115 (35)	501 (25)	616 (26)	<0.001
NS5A ± NS3/4A protease inhibitor	15 (5)	98 (5)	113 (5)	0.857
Fibrosis stage, n (%)^c^				
F0–F1	188 (57)	1463 (72)	1651 (70)	<0.001
F2	33 (10)	132 (6)	165 (7)	0.018
F3	45 (14)	200 (10)	245 (10)	0.032
F4	62 (19)	241 (12)	303 (13)	<0.001
Cirrhosis status, n (%)				
Compensated cirrhosis	64 (20)	244 (12)	308 (13)	<0.001
No cirrhosis	264 (80)	1797 (88)	2061 (87)	<0.001
Estimated glomerular filtration rate <30 mL/min/1.73 m^2^, n (%)^d^	28 (9)	75 (4)	103 (5)	<0.001
Diabetes, n (%)	57 (17)	147 (7)	204 (9)	<0.001
Bipolar disorder or depression, n (%)	52 (16)	456 (22)	508 (21)	0.008
Hypertension, n (%)	177 (54)	478 (23)	655 (28)	<0.001
Cardiovascular disease, n (%)	204 (62)	572 (28)	776 (33)	<0.001
Glecaprevir/pibrentasvir treatment duration, n (%)				
8 weeks	94 (29)	756 (37)	850 (36)	0.003
12 weeks	214 (65)	1185 (58)	1399 (59)	0.014
16 weeks	20 (6)	100 (5)	120 (5)	0.358
Treatment compliance^e^, n (%)				
Compliant	289 (88)	1832 (90)	2121 (90)	0.365
Non-compliant	39 (12)	209 (10)	248 (10)	0.365

GT, genotype; HCV, hepatitis C virus; *IL28B*, interleukin 28B; PegIFN, pegylated interferon; RBV, ribavirin; RNA, ribonucleic acid; SD, standard deviation.

^a^Other category includes American Indian or Alaska native, Native Hawaiian or other Pacific Islander, multiple races, and missing data.

^b^Data missing for 1 patient each from the elderly and non-elderly cohorts.

^c^Data missing for 5 patients in the non-elderly cohort.

^d^Data missing for 1 patient in the non-elderly cohort; overall percentage calculated using a total population (N) of 2238.

^e^Compliant was defined as 80%–120% of expected glecaprevir/pibrentasvir intake.

^f^*P*-values were calculated for the elderly and non-elderly comparison using a Chi-square test for categorical data and one-way ANOVA for continuous data; *P*-values for treatment compliance are based on 2 by 2 contingency table.

**Table 2:** Concomitant medications.

Medication, n (%)	Patients Aged ≥65 Years (n = 328)	Patients Aged <65 Years (n = 2041)	Total (N = 2369)	*P*-value^c^
Any	302 (92)	1638 (80)	1940 (82)	<0.001
Acid-reducing agents^a^	63 (19)	299 (15)	362 (15)	0.332
Antidepressants	46 (14)	317 (16)	363 (15)	0.482
Antihypertensives^b^	146 (45)	364 (18)	510 (22)	<0.001
Diuretics	50 (15)	131 (6)	181 (8)	<0.001
Antipsychotics	10 (3)	88 (4)	98 (4)	0.286
Diabetes medications (including insulin)	50 (15)	145 (7)	195 (8)	<0.001
Lipid-lowering drugs	49 (15)	134 (7)	183 (8)	<0.001

^a^Includes antacids and proton pump inhibitors.

^b^Includes angiotensin II antagonists, beta-blocking drugs, calcium channel blockers, potassium-sparing drugs, and angiotensin-converting enzyme inhibitors (patients may have been receiving more than 1 of these medications and therefore may have been counted more than once).

^c^*P*-values were calculated using a Chi-square test.

### Efficacy

**Fig 1:**
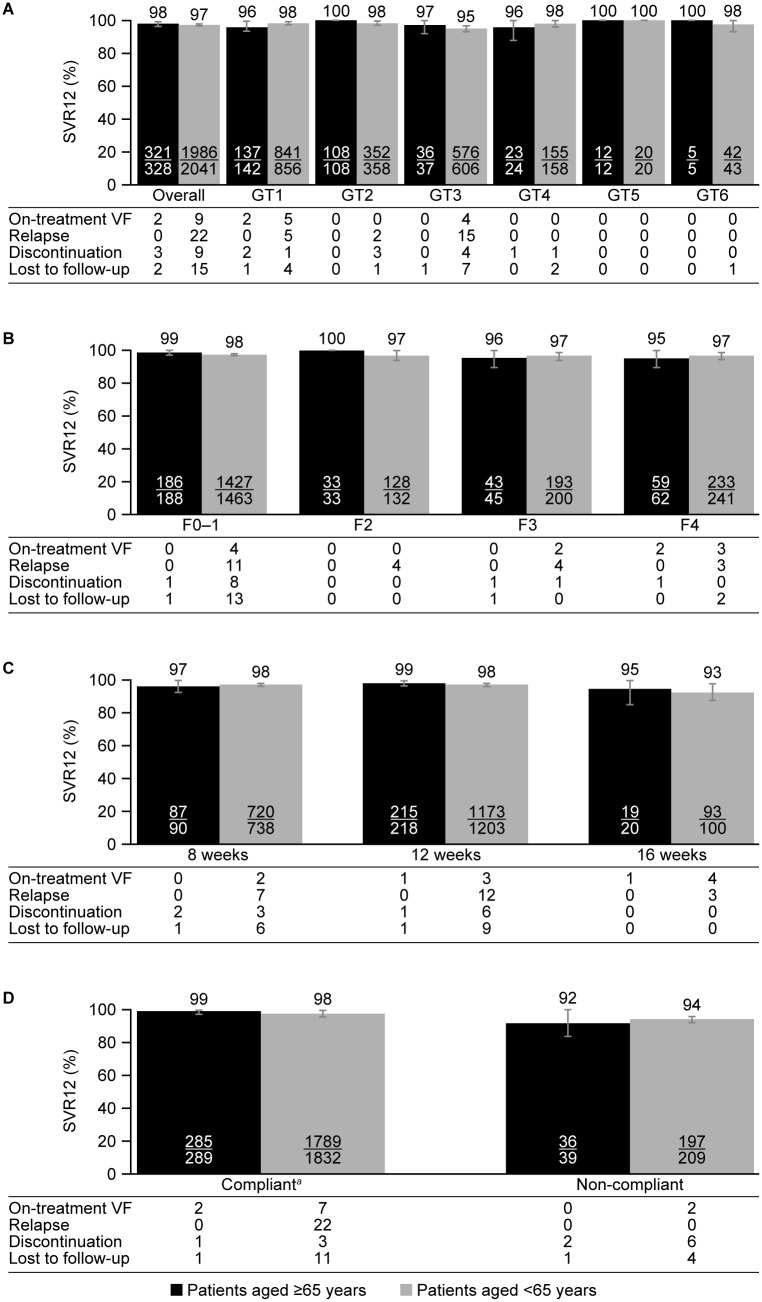
Sustained virologic response at post-treatment week 12 by A) hepatitis C virus genotype; B) fibrosis stage; C) glecaprevir/pibrentasvir treatment duration; and D) glecaprevir/pibrentasvir treatment compliance^a^ (ITT analyses). ^a^Compliant was defined as 80%–120% of expected glecaprevir/pibrentasvir intake. F, fibrosis stage; GT, genotype; ITT, intention-to-treat; SVR12, sustained virologic response at post-treatment week 12; VF, virologic failure.

The overall SVR12 rate for the ITT population was 97.4% (95% CI, 96.7–98.0; n/N = 2307/2369). Elderly patients achieved an SVR12 rate of 97.9% (95% CI, 96.3–99.4; n/N = 321/328) and the non-elderly patients achieved an SVR12 rate of 97.3% (95% CI, 96.6–98.0; n/N = 1986/2041) (Fig 1A). The rates were not significantly different between the two age groups (*P* = 0.555). The overall SVR12 rate for the mITT population was 98.6% (95% CI, 98.1–99.1; n/N = 2307/2340): 99.4% (95% CI, 98.5–100; n/N = 321/323) for elderly patients and 98.5% (95% CI, 97.9–99.0; n/N = 1986/2017) for non-elderly patients.

Overall SVR12 rates were generally unaffected by HCV GT, fibrosis stage, treatment duration and compliance (Fig 1A–1D; S3 Table). SVR12 rates were similar for elderly and non-elderly patients across HCV GTs, fibrosis stages, and glecaprevir/pibrentasvir treatment durations (Fig 1A–1C; S3 Table). SVR12 rates also appeared to be comparable between elderly and non-elderly patients grouped by treatment compliance status (Fig 1D; S3 Table).

Seven elderly patients (2%) did not achieve SVR12 (mean [standard deviation] age, 67 [±1.7] years). Of these, 3 patients discontinued treatment, 2 patients had on-treatment virologic failure, and 2 patients had missing SVR12 data.

### Safety

The overall safety profile of elderly patients without severe renal impairment was similar to that of non-elderly patients (Table 3). A total of 63% of elderly patients without severe renal impairment experienced AEs, most of which were Grade 1 (mild) to Grade 2 (moderate) in severity. The most common AEs experienced by elderly patients without severe renal impairment were headache (12%) and fatigue (11%). There were some statistically significant differences between elderly and non-elderly patients in the frequency of individual AEs and the incidence of serious AEs; however, the small sample sizes in these subsets of patients make it difficult to achieve clinically meaningful conclusions. AEs were considered to be related to glecaprevir/pibrentasvir treatment in 36% of elderly patients. Four percent of elderly patients without severe renal impairment experienced serious AEs (SAEs), none of which were considered to be related to glecaprevir/pibrentasvir treatment. None of the elderly patients discontinued treatment because of AEs related to glecaprevir/pibrentasvir treatment. Less than 1% of patients without severe renal impairment died: 2 patients in the elderly cohort and 4 in the non-elderly cohort. None of the deaths were considered to be related to glecaprevir/pibrentasvir treatment; MedDRA-coded preferred terms listed for cause of death were: metastatic hepatic cancer, pneumonia, death, accidental overdose, cerebral hemorrhage, adenocarcinoma.

**Table 3:** Summary of adverse events for patients without severe renal impairment^a^.

Event, n (%)	Patients Aged ≥65 Years (n = 300)	Patients Aged <65 Years (n = 1965)	*P*-value^b^
Any AE	189 (63)	1340 (68)	0.074
AEs experienced by ≥10% of patients			
Headache	36 (12)	374 (19)	0.003
Fatigue	32 (11)	298 (15)	0.04
Nausea	18 (6)	190 (10)	0.04
Any DAA-related AE	107 (36)	822 (42)	0.043
Any AE with Grade 3 severity or greater	13 (4)	52 (3)	0.103
Any DAA-related AE with Grade 3 severity or greater	0	4 (<1)	0.434
Any serious AE	13 (4)	35 (2)	0.004
Any DAA-related serious AE	0	1 (<1)	0.696
Any AE leading to treatment discontinuation	2 (<1)	6 (<1)	0.326
Any DAA-related AE leading to treatment discontinuation	0	3 (<1)	0.498
Fatal AEs	1 (<1)	1 (<1)	0.125
Deaths	2 (<1)	4 (<1)	0.146

AE, adverse event; DAA, direct-acting antiviral.

^a^Estimated glomerular filtration rate ≥30 mL/min/1.73 m^2^.

^b^*P*-values were calculated for the elderly and non-elderly comparison using a Chi-square test.

For patients with severe renal impairment, 79% of elderly and 68% of non-elderly patients experienced AEs (Table 4). The most common AE experienced by elderly patients with severe renal impairment was pruritus (32%) followed by fatigue, nausea, asthenia, and decreased appetite (18% each), which are consistent with the underlying renal failure. The AEs among these patients were considered to be treatment related in 57% of elderly and 46% of non-elderly patients. A total of 39% of elderly patients experienced AEs of Grade ≥3 severity compared with 18% of non-elderly patients. Grade ≥3 AEs were considered to be treatment related in 7% and 4% of elderly and non-elderly patients with severe renal impairment, respectively. A total of 39% of elderly patients with severe renal impairment experienced SAEs, of which none were considered to be treatment related. One patient with severe renal impairment discontinued treatment because of AEs that were considered to be related to glecaprevir/pibrentasvir treatment. One non-elderly patient with severe renal impairment died; the death was not considered related to glecaprevir/pibrentasvir treatment.

**Table 4:** Summary of adverse events for patients with severe renal impairment^a^.

Event, n (%)	Patients Aged ≥65 Years (n = 28)	Patients Aged <65 Years (n = 76)	*P*-value^b^
Any AE	22 (79)	52 (68)	0.311
AEs experienced by ≥10% of patients^c^			
Fatigue	5 (18)	10 (13)	
Nausea	5 (18)	7 (9)	
Diarrhea	4 (14)	6 (8)	
Pruritus	9 (32)	12 (16)	
Asthenia	5 (18)	5 (7)	
Decreased appetite	5 (18)	4 (5)	
Abdominal pain	3 (11)	1 (1)	
Any DAA-related AE	16 (57)	35 (46)	0.316
Any AE with Grade 3 severity or greater	11 (39)	14 (18)	0.027
Any DAA-related AE with Grade 3 severity or greater	2 (7)	3 (4)	0.499
Any serious AE	11 (39)	14 (18)	0.027
Any DAA-related serious AE	0	0	
Any AE leading to treatment discontinuation	2 (7)	2 (3)	0.289
Any DAA-related AE leading to treatment discontinuation	1 (4)	1 (1)	0.458
Fatal AEs	0	1 (1)	0.542
Deaths	0	1 (1)	0.542

AE, adverse event; DAA, direct-acting antiviral.

^a^Estimated glomerular filtration rate <30 mL/min/1.73 m^2^.

^b^*P-*values were calculated for the elderly and non-elderly comparison using a Chi-square test.

^c^*P-*values not calculated due to small sample size (<10) in either group.

Few elderly patients experienced clinically significant laboratory abnormalities (Table 5). Overall, <1% (2 patients) had Grade 3 elevations in total bilirubin levels, <1% (1 patient) had Grade 3 decreases in hemoglobin levels, and <1% (1 patient) had Grade 3 decreases in platelet levels. Post-baseline laboratory abnormalities were similarly infrequent (1%) in non-elderly patients.

**Table 5:** Summary of post-baseline laboratory abnormalities.

Event, n/N (%)	Patients Aged ≥65 Years	Patients Aged <65 Years
Alanine aminotransferase		
Grade 3	0	2/2039 (<1)^a^^,^^b^
Grade 4	0	0
Total bilirubin		
Grade 3	2/328 (<1)	7/2039 (<1)^b^
Grade 4	0	0

^a^The laboratory results were not associated with drug-induced liver injury, but were instead consistent with fluctuations in alanine aminotransferase during the first 2 weeks of treatment or were due to other etiologies, such as gallstones.

^b^One patient with concurrent Grade 3 alanine aminotransferase and total bilirubin changes, which were attributable to gallstones.

## Discussion

This retrospective analysis of pooled data from 9 Phase 2 and 3 clinical trials was conducted to assess the safety and efficacy of combination glecaprevir/pibrentasvir in elderly patients (aged 65 years or older). The results of this analysis indicate that once-daily glecaprevir/pibrentasvir for 8, 12, or 16 weeks is a safe, well-tolerated, and highly efficacious treatment for chronic HCV infection in elderly patients. The efficacy of glecaprevir/pibrentasvir was not significantly impacted by HCV GT, fibrosis stage, treatment duration, or compliance, and was similar to the efficacy observed in younger patients in these trials.

Glecaprevir/pibrentasvir was generally well tolerated by the elderly patients. Most AEs were Grade 1 (mild) to Grade 2 (moderate) in severity, no SAEs were considered to be related to treatment, and treatment discontinuations assessed as related to glecaprevir/pibrentasvir were rare (<1%). Elderly patients with severe renal impairment tended to have a greater incidence of some types of AEs; however, most AEs were considered unrelated to treatment with glecaprevir/pibrentasvir, and the greater incidence of AEs experienced by this subgroup of patients is expected given the severe renal impairment and the associated comorbidities [24, 25]. For patients without severe renal impairment, the safety profiles of older and younger patients were comparable.

The findings of this analysis add to the existing results obtained from previous studies of DAAs for the treatment of chronic HCV infection in elderly patients. In a retrospective analysis of pooled data from 4 Phase 3 clinical trials, ledipasvir/sofosbuvir with and without RBV for 8, 12, or 24 weeks resulted in high efficacy for patients with chronic HCV GT1 infections aged ≥65 years (SVR12 rate, 98% [95% CI, 95–99]; n = 264) and for patients aged <65 years (SVR12 rate, 97% [95% CI, 96–98]; n = 2029) [13]. Although elderly patients had a greater prevalence of cirrhosis at baseline, the efficacy of ledipasvir/sofosbuvir was not impacted, which is consistent with our results [13].

Data accumulating from real-world clinical practice also support the high efficacy and safety of all-oral DAAs reported in clinical trials. Elderly patients aged ≥65 years in Italy with chronic HCV GT1–4 infections and advanced fibrosis or cirrhosis who received a variety of sofosbuvir- and ombitasvir/paritaprevir/ritonavir-containing regimens, achieved an SVR12 rate of 94.7% (n = 282) versus 90.5% for patients aged <65 years (n = 274) [9]. SAEs were uncommon (5%) and treatment discontinuations were rare (2%) in elderly patients compared with younger patients, despite a greater prevalence of liver cancer history, more severe liver disease, and presence of comorbidities such as arterial hypertension and renal disease [9]. In the large real-world Spanish National Registry (Hepa-C) of >1200 patients aged ≥65 years with chronic HCV GT1–5 infections treated with all-oral DAA regimens (mostly ledipasvir/sofosbuvir-, simeprevir/sofosbuvir-, and ombitasvir/paritaprevir/ritonavir-containing regimens), the SVR12 rate was 94% [10]. Likewise, in a real-world analysis of >17,000 patients in the US Veterans Affairs Healthcare System (>4500 patients aged ≥65 years) with GT1–4 infections treated with ledipasvir/sofosbuvir- and ombitasvir/paritaprevir/ritonavir-containing regimens, advanced age was found to be not significantly associated with the likelihood of achieving SVR12 [12].

In addition to clinical trials and real-world efficacy and safety studies of DAAs, emerging data suggest that successful antiviral treatment in elderly patients can improve life expectancy and health-related quality of life [26, 27]. These data suggest that the benefits of treating chronic HCV infection in elderly patients may go beyond the elimination of HCV and HCV infection-related comorbidities.

A key strength of this pooled analysis is that it included data from 9 Phase 2 and 3 well-defined clinical trials with all available clinical and biological data, to assess the safety and efficacy of combination glecaprevir/pibrentasvir in elderly patients (aged 65 years or older). The results of this analysis indicate that once-daily glecaprevir/pibrentasvir for 8, 12, or 16 weeks is a well-tolerated and highly efficacious treatment for chronic HCV infection in elderly patients. Furthermore, eligibility criteria for the trials in this analysis specified no upper age limit for inclusion; therefore, the study population represents a broad group of elderly patients. Limitations include that this meta-analysis was *post hoc*. The clinical trials included in this analysis were not designed or statistically powered to compare specifically the efficacy and safety of glecaprevir/pibrentasvir in the elderly versus non-elderly patients.

In conclusion, glecaprevir/pibrentasvir is efficacious and well tolerated for the treatment of chronic HCV infections in elderly patients. These findings add to the growing body of clinical trial and real-world data supporting that age should not be a barrier to the initiation and successful treatment of chronic HCV infection.

## Supporting information

S1 Table Concomitant medications for patients with severe renal impairment. ^a^Includes antacids and proton pump inhibitors. ^b^Includes angiotensin II antagonists, beta-blocking drugs, calcium channel blockers, potassium-sparing drugs, and angiotensin-converting enzyme inhibitors (patients may have been receiving more than 1 of these medications and therefore may have been counted more than once). ^c^Includes combination diuretics and potassium-sparing drugs, high-ceiling diuretics, low-ceiling diuretics (excluding thiazides), low-ceiling diuretics (thiazides), and other diuretics. ^d^*P*-values were calculated for the elderly and non-elderly comparison using a Chi-square test.
(DOCX)

S2 Table Treatment compliance rates for patients receiving 4 or more concomitant medications^a^. ^a^In total (including glecaprevir/pibrentasvir), patients were receiving 5 or more concomitant medications; cut-off based on Masnoon N, et al. *BMC Geriatr*. 2017;17: 230; https://www.ncbi.nlm.nih.gov/pmc/articles/pmc5635569/. ^b^95% confidence intervals calculated using the normal approximation to the binomial distribution. ^c^*P* = 0.045 for the difference between the age groups (using a Chi-square test).
(DOCX)

S3 Table Sustained virologic responses at post-treatment week 12 by hepatitis C virus genotype (mITT analysis). CI, confidence interval; GT, genotype; HCV, hepatitis C virus; mITT, modified intention-to-treat; SVR12, sustained virologic response at post-treatment week 12. ^a^*P*-values were calculated for the elderly and non-elderly comparison using a Chi-square test.
(DOCX)

## References

[1] Denniston MM, Jiles RB, Drobeniuc J, Klevens RM, Ward JW, McQuillan GM, et al Chronic hepatitis C virus infection in the United States, National Health and Nutrition Examination Survey 2003 to 2010. Ann Intern Med. 2014;160: 293–300. 10.7326/M13-1133 24737271 PMC4562398

[2] Smith BD, Morgan RL, Beckett GA, Falck-Ytter Y, Holtzman D, Ward JW. Hepatitis C virus testing of persons born during 1945–1965: recommendations from the Centers for Disease Control and Prevention. Ann Intern Med. 2012;157: 817–22. 10.7326/0003-4819-157-9-201211060-00529 22910836 PMC5777166

[3] Razavi H, Waked I, Sarrazin C, Myers RP, Idilman R, Calinas F, et al The present and future disease burden of hepatitis C virus (HCV) infection with today’s treatment paradigm. J Viral Hepat. 2014;21 Suppl 1: 34–59. 10.1111/jvh.12248 24713005

[4] Chung H, Ueda T, Kudo M. Changing trends in hepatitis C infection over the past 50 years in Japan. Intervirology. 2010;53: 39–43. 10.1159/000252782 20068339

[5] Sagnelli E, Stroffolini T, Sagnelli C, Cacopardo B, Andriulli A, Babudieri S, et al Characteristics of patients with hepatitis C virus-related chronic liver diseases just before the era of oral direct-acting antiviral therapy in Italy. Eur J Gastroenterol Hepatol. 2018;30: 676–81. 29465473 10.1097/MEG.0000000000001099

[6] Saab S, Rheem J, Sundaram V. Hepatitis C Infection in the Elderly. Dig Dis Sci. 2015;60: 3170–80. 10.1007/s10620-015-3717-6 26008618

[7] Huang CF, Yu ML. Treating hepatitis C in the elderly: pharmacotherapeutic considerations and developments. Expert Opin Pharmacother. 2017;18: 1867–74. 10.1080/14656566.2017.1400010 29086615

[8] Vermehren J, Peiffer KH, Welsch C, Grammatikos G, Welker MW, Weiler N, et al The efficacy and safety of direct acting antiviral treatment and clinical significance of drug-drug interactions in elderly patients with chronic hepatitis C virus infection. Aliment Pharmacol Ther. 2016;44: 856–65. 10.1111/apt.13769 27549000

[9] Conti F, Brillanti S, Buonfiglioli F, Vukotic R, Morelli MC, Lalanne C, et al Safety and efficacy of direct-acting antivirals for the treatment of chronic hepatitis C in a real-world population aged 65 years and older. J Viral Hepat. 2017;24: 454–63. 10.1111/jvh.12663 27976461

[10] Lens S, Fernandez I, Rodriguez-Tajes S, Hontangas V, Vergara M, Forne M, et al Interferon-Free Therapy in Elderly Patients With Advanced Liver Disease. Am J Gastroenterol. 2017;112: 1400–9. 10.1038/ajg.2017.157 28585554

[11] Rodriguez-Osorio I, Cid P, Morano L, Castro A, Suarez M, Delgado M, et al Real life experience with direct-acting antivirals agents against hepatitis C infection in elderly patients. J Clin Virol. 2017;88: 58–61. 10.1016/j.jcv.2017.01.003 28183063

[12] Su F, Beste LA, Green PK, Berry K, Ioannou GN. Direct-acting antivirals are effective for chronic hepatitis C treatment in elderly patients: a real-world study of 17 487 patients. Eur J Gastroenterol Hepatol. 2017;29: 686–93. 10.1097/MEG.0000000000000858 28195877 PMC6534142

[13] Saab S, Park SH, Mizokami M, Omata M, Mangia A, Eggleton E, et al Safety and efficacy of ledipasvir/sofosbuvir for the treatment of genotype 1 hepatitis C in subjects aged 65 years or older. Hepatology. 2016;63: 1112–9. 10.1002/hep.28425 26704693

[14] Reau N, Kwo PY, Rhee S, Brown RS Jr., Agarwal K, Angus P, et al Glecaprevir/Pibrentasvir Treatment in Liver or Kidney Transplant Patients With Hepatitis C Virus Infection. Hepatology. 2018 10.1002/hep.30046 29672891 PMC6220874

[15] Asselah T, Kowdley KV, Zadeikis N, Wang S, Hassanein T, Horsmans Y, et al Efficacy of Glecaprevir/Pibrentasvir for 8 or 12 Weeks in Patients With Hepatitis C Virus Genotype 2, 4, 5, or 6 Infection Without Cirrhosis. Clin Gastroenterol Hepatol. 2018;16: 417–26. 10.1016/j.cgh.2017.09.027 28951228

[16] Forns X, Lee SS, Valdes J, Lens S, Ghalib R, Aguilar H, et al Glecaprevir plus pibrentasvir for chronic hepatitis C virus genotype 1, 2, 4, 5, or 6 infection in adults with compensated cirrhosis (EXPEDITION-1): a single-arm, open-label, multicentre phase 3 trial. Lancet Infect Dis. 2017;17: 1062–8. 10.1016/S1473-3099(17)30496-6 28818546

[17] Gane E, Lawitz E, Pugatch D, Papatheodoridis G, Brau N, Brown A, et al Glecaprevir and Pibrentasvir in Patients with HCV and Severe Renal Impairment. N Engl J Med. 2017;377: 1448–55. 10.1056/NEJMoa1704053 29020583

[18] Gane E, Poordad F, Wang S, Asatryan A, Kwo PY, Lalezari J, et al High efficacy of ABT-493 and ABT-530 treatment in patients with HCV genotype 1 or 3 infection and compensated cirrhosis. Gastroenterology. 2016;151: 651–9 e1. 10.1053/j.gastro.2016.07.020 27456384

[19] Kwo PY, Poordad F, Asatryan A, Wang S, Wyles DL, Hassanein T, et al Glecaprevir and pibrentasvir yield high response rates in patients with HCV genotype 1–6 without cirrhosis. J Hepatol. 2017;67: 263–71. 10.1016/j.jhep.2017.03.039 28412293

[20] Poordad F, Felizarta F, Asatryan A, Sulkowski MS, Reindollar RW, Landis CS, et al Glecaprevir and pibrentasvir for 12 weeks for hepatitis C virus genotype 1 infection and prior direct-acting antiviral treatment. Hepatology. 2017;66: 389–97. 10.1002/hep.29081 28128852 PMC5573922

[21] Wyles D, Poordad F, Wang S, Alric L, Felizarta F, Kwo PY, et al Glecaprevir/pibrentasvir for hepatitis C virus genotype 3 patients with cirrhosis and/or prior treatment experience: A partially randomized phase 3 clinical trial. Hepatology. 2017 10.1002/hep.29541 28926120 PMC5817409

[22] Poordad F, Pol S, Asatryan A, Buti M, Shaw D, Hezode C, et al Glecaprevir/Pibrentasvir in patients with hepatitis C virus genotype 1 or 4 and past direct-acting antiviral treatment failure. Hepatology. 2018;67: 1253–60. 10.1002/hep.29671 29152781 PMC5901397

[23] Zeuzem S, Foster GR, Wang S, Asatryan A, Gane E, Feld JJ, et al Glecaprevir-Pibrentasvir for 8 or 12 Weeks in HCV Genotype 1 or 3 Infection. N Engl J Med. 2018;378: 354–69. 10.1056/NEJMoa1702417 29365309

[24] Combs SA, Teixeira JP, Germain MJ. Pruritus in Kidney Disease. Semin Nephrol. 2015;35: 383–91. 10.1016/j.semnephrol.2015.06.009 26355256 PMC5497472

[25] Kidney Disease: Improving Global Outcomes (KDIGO) CKD Work Group. KDIGO 2012 Clinical Practice Guideline for the Evaluation and Management of Chronic Kidney Disease. Kidney International Supplements. 2013;3: 1–150. 10.1016/j.kisu.2017.10.001 PMC6341011 30681074

[26] Maor Y, Malnick SD, Melzer E, Leshno M. Treatment of Chronic Hepatitis C in the Aged—Does It Impact Life Expectancy? A Decision Analysis. PLoS One. 2016;11: e0157832 10.1371/journal.pone.0157832 27410963 PMC4943667

[27] Younossi ZM, Stepanova M, Nader F, Henry L. Patient-Reported Outcomes of Elderly Adults with Chronic Hepatitis C Treated with Interferon- and Ribavirin-Free Regimens. J Am Geriatr Soc. 2016;64: 386–93. 10.1111/jgs.13928 26825683

