# Supplementary material for: Safety and efficacy of glecaprevir/pibrentasvir for the treatment of chronic hepatitis C in patients aged 65 years or older
Source: PLoS One. 2019 Jan 2;14(1):e0208506. doi: 10.1371/journal.pone.0208506 (PMC6314565; doi:10.1371/journal.pone.0208506)
Supplement: S1 Table — aIncludes antacids and proton pump inhibitors. bIncludes angiotensin II antagonists, beta-blocking drugs, calcium channel blockers, potassium-sparing drugs, and angiotensin-converting enzyme inhibitors (patients may have been receiving more than 1 of these medications and therefore may have been counted more than once). cIncludes combination diuretics and potassium-sparing drugs, high-ceiling diuretics, low-ceiling diuretics (excluding thiazides), low-ceiling diuretics (thiazides), and other diuretics. dP-values were calculated for the elderly and non-elderly comparison using a Chi-square test. (DOCX) [file pone.0208506.s001.docx]

**S1 Table. Concomitant Medications for Patients With Severe Renal Impairment.**

| **Medication, n (%)** | **Patients Aged ≥65 Years  (n = 28)** | **Patients Aged <65 Years  (n = 76)** |
| --- | --- | --- |
| Any | 28 (100) | 76 (100) |
| Acid-reducing agents^a^ | 11 (39) | 37 (49) |
| Antidepressants | 3 (11) | 7 (9) |
| Antihypertensives^b^ | 19 (68) | 59 (78) |
| Antipsychotics | 1 (4) | 2 (3) |
| Diuretics^c^ | 12 (43) | 22 (29) |
| Lipid-lowering drugs | 10 (36) | 14 (18) |

^a^Includes antacids and proton pump inhibitors.

^b^Includes angiotensin II antagonists, beta-blocking drugs, calcium channel blockers, potassium-sparing drugs, and angiotensin-converting enzyme inhibitors (patients may have been receiving more than 1 of these medications and therefore may have been counted more than once).

^c^Includes combination diuretics and potassium-sparing drugs, high-ceiling diuretics, low-ceiling diuretics (excluding thiazides), low-ceiling diuretics (thiazides), and other diuretics.
