# Supplementary material for: Safety and efficacy of glecaprevir/pibrentasvir for the treatment of chronic hepatitis C in patients aged 65 years or older
Source: PLoS One. 2019 Jan 2;14(1):e0208506. doi: 10.1371/journal.pone.0208506 (PMC6314565; doi:10.1371/journal.pone.0208506)
Supplement: S2 Table — aIn total (including glecaprevir/pibrentasvir), patients were receiving 5 or more concomitant medications; cut-off based on Masnoon N, et al. BMC Geriatr. 2017;17: 230; https://www.ncbi.nlm.nih.gov/pmc/articles/pmc5635569/. b95% confidence intervals calculated using the normal approximation to the binomial distribution. cP = 0.045 for the difference between the age groups (using a Chi-square test). (DOCX) [file pone.0208506.s002.docx]

**S2 Table.** **Sustained Virologic Responses at Post-treatment Week 12 by Hepatitis C Virus Genotype (mITT Analysis).**

| **SVR12, % (95% CI; n/N)** | **Patients Aged ≥65 Years  (n = 323)** | **Patients Aged <65 Years  (n = 2017)** |
| --- | --- | --- |
| Overall | 99.4 (98.5–100; 321/323) | 98.5 (97.9–99.0; 1986/2017) |
| HCV genotype  GT1  GT2  GT3  GT4  GT5  GT6 | 98.6 (96.6–100; 137/139)  100 (100–100; 108/108)  100 (100–100; 36/36)  100 (100–100; 23/23)  100 (100–100; 12/12)  100 (100–100; 5/5) | 98.8 (98.1–99.5; 841/851)  99.4 (98.7–100; 352/354)  96.8 (95.4–98.2; 576/595)  100 (100–100; 155/155)  100 (100–100; 20/20)  100 (100–100; 42/42) |
| Fibrosis stage  F0–F1  F2  F3  F4 | 100 (100–100; 186/186)  100 (100–100; 33/33)  100 (100–100; 43/43)  96.7 (92.3–100; 59/61) | 99.0 (98.4–99.5; 1427/1442)  97.0 (94.0–99.9; 128/132)  97.0 (94.6–99.4; 193/199)  97.5 (95.5–99.5; 233/239) |
| Glecaprevir/pibrentasvir-treatment compliance  Compliant  Non-compliant | 99.3 (98.3–100; 285/287)  100 (100–100; 36/36) | 98.4 (97.8–99.0; 1789/1818)  99.0 (97.6–100; 197/199) |

CI, confidence interval; GT, genotype; HCV, hepatitis C virus; mITT, modified intention-to-treat; SVR12, sustained virologic response at post-treatment week 12.
